# Study on the Phase Equilibria of the Fe-Al-Ni-O System at 750°C

**DOI:** 10.1155/2022/4188727

**Published:** 2022-01-15

**Authors:** Meng Du, Haifeng Mei, Ya Liu

**Affiliations:** ^1^College of Vehicle Engineering, Changzhou Vocational Institute of Mechatronic Technology, Changzhou 213164, China; ^2^School of Materials Science and Engineering, Changzhou University, Changzhou 213164, China; ^3^Jiangsu Key Laboratory of Materials Surface Science and Technology, Changzhou University, Changzhou 213164, China

## Abstract

Phase equilibria of the Fe-Al-Ni-O system at 750°C were determined by scanning electron microscopy coupled with energy-dispersive X-ray spectrometer and X-ray power diffraction. 54 alloys were prepared with weighted metal and Ni_2_O_3_ powder and were annealed at 750°C for 45 days. Two four-phase equilibrium regions and three three-phase equilibrium regions were confirmed, and the boundary between spinel and corundum was obtained. Comparing with the Fe-Al-Ni-O oxidation diagram at 750°C calculated with FSstel and FToxid databases, the phase boundary of the spinel and corundum oxides from experiments was inclined to the Ni-Al side. The determined relationship between primary oxides and alloy composition in this work can be used as a reference for the preparation of the oxide film by selective oxidation.

## 1. Introduction

Corrosion is one of the major problems hindering the development and industrial application of steel. Anticorrosion surface protective layer has been prepared not only by dipping, infiltrating, or injecting more than one kind of anticorrosion element or compound on the surface of metal material but also by high-temperature preoxidation treatment [1–6]. If enough Cr, Al, Si, and Ti are added into the alloy, oxide film such as Cr_2_O_3_, Al_2_O_3_, SiO_2_, and spinel will be formed. These oxides have less defects, compactness, good adhesion, and high temperature stability, which can effectively prevent corrosion and protect the alloy [1, 4, 5, 7].

Under the condition of high temperature and low oxygen pressure, the main elements (Fe, Ni, and Co) of the alloy will not be oxidized, but the elements (Cr, Al, Si, and Ti) with high affinity to oxygen in the alloy are easy to be oxidized according to the Ellingham diagram [8]. Selective oxidation promotes the enrichment of alloy elements to the surface, and only a small amount of alloy elements can form a complete and dense oxide film [3–7], so the addition of alloy elements is greatly reduced, which makes the design of materials more flexible.

Alloy composition has a great influence on oxidation. There are many studies on the oxidation and wettability of Fe-Ni-based alloys and the oxidation behavior of other alloys in air in the literature [9–11]. However, there are few systematic studies on the influence of alloy composition change on the oxidation process at low oxygen pressure based on the development of anticorrosion oxide film. The relationship between primary oxide and alloy composition is called primary oxidation phase diagram, which is an important basis for understanding the effect of alloy composition change on oxidation process. Our research group has carried out some work in this area [12, 13].

Rhamdhani et al. [14] had researched the subsolidus phase equilibrium of Fe-Al-Ni-O system in air. Raghavan [15] had also discussed equilibria of oxide phases at high temperature in the atmosphere. However, there are relatively few researches on the phase equilibrium of the Fe-Al-Ni-O system under the circumstance of low oxygen content. In this paper, the phase relationship of the Fe-Al-Ni-O system at 750°C was determined by thermodynamic calculation and experiments, which would be helpful to explain the selective oxidation of iron-based or nickel-based alloys with Al addition.

## 2. Literature Data

The four constituting ternaries of the Fe-Al-Ni-O quaternary are the Fe-Al-Ni, Fe-Al-O, Fe-Ni-O, and Al-Ni-O systems. Crystal structure data for the matrix phases and oxides in the Fe-Al-Ni-O system is summarized in Table 1.

### 2.1. The Fe-Al-Ni Ternary System

**Figure 1:**
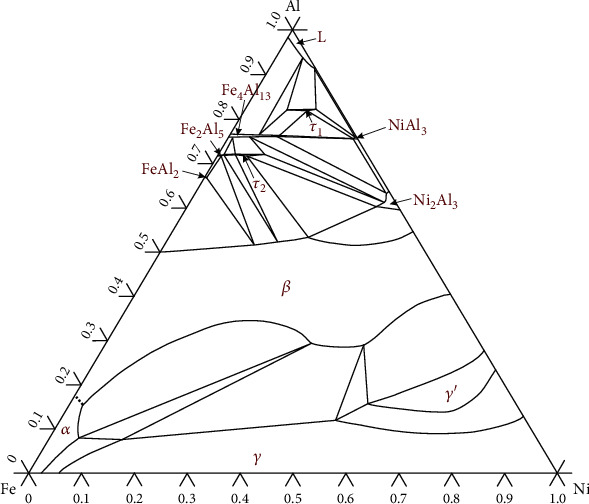
The calculated 750°C isothermal section of the Fe-Al-Ni ternary system.

The Fe-Al-Ni ternary system has been studied experimentally, evaluated thermodynamically, reviewed, and updated many times. The most recent review was published by Raghavan [17] in 2010, in which the work from Zhang and Du [18] was presented. The isothermal section of the Fe-Al-Ni ternary system at 750°C shown in Figure 1 was redrawn based on thermodynamic description from Zhang and Du [18]. There are four solid solution phases (namely, ɑ, *β*, *γ*, and *γ*′), five binary intermetallic compounds (FeAl_2_, Fe_2_Al_5_, Fe_4_Al_13_, NiAl_3_, and Ni_2_Al_3_), and two ternary intermetallic compounds (*τ*_1_ and *τ*_2_) included in this isothermal section.

### 2.2. The Fe-Al-O System

Raghavan reviewed the Fe-Al-O system twice. The recent article in 2010 presented the Al-Fe-O pseudobinary section in air along the Fe_2_O_3_-Al_2_O_3_ join [19]. Since then, Lindwall et al. [20] and Shishin et al. [21] assessed this system thermodynamically in 2015 and 2016, respectively. The following oxidation phases, corundum (Al_2_O_3_ and Fe_2_O_3_), wustite (FeO), magnetite (Fe_3_O_4_), and spinel (FeAl_2_O_4_), were included in the Al-Fe-O system.

### 2.3. The Fe-Ni-O System

Raghavan [22] reviewed the research results on the Fe-Ni-O system. In addition to the calculated Fe-Ni-O isothermal section at 1540°C by Luoma [23], this review had also presented Fe-Ni-O pseudobinary section along the Fe_2_O_3_-NiO join in air and the Fe-Ni-O isothermal phase diagram at low oxygen partial pressure between 1000°C and 1200°C [24]. NiFe_2_O_4_, NiO, FeO, Fe_2_O_3_, and Fe_3_O_4_ oxidation phases were evidenced in these isothermal sections.

### 2.4. The Al-Ni-O System

As early as 1981, Elrefaie and Smeltzer [25] determined the equilibrium oxygen pressure of Ni-NiO-NiAl_2_O_4_ and Ni-NiAl_2.54_O_4.81_-Al_2_O_3_ systems between 850°C and 1050°C by EMF method; Saltykov et al. [26] thermodynamically evaluated the Ni-Al-O system. Al_2_O_3_, NiAl_2_O_4_, NiO, Ni_2_O_3_, and Ni_3_O_4_ were stable in the 940°C isothermal section of the Al-Ni-O system. However, there are few reports on the phase equilibrium of the Ni-Al-O system since then, probably due to the low oxygen partial pressure.

### 2.5. The Fe-Al-Ni-O System

Information on the phase equilibrium of the Fe-Al-Ni-O system is scary, except for the results reported by Rhamdhani et al. [14] and Kjellqvist et al. [27]. Three three-phase equilibria of spinel+corundum+hematite, spinel+corundum+Fe_2_Al_2_O_6_, and spinel+hematite+Fe_2_Al_2_O_6_ were found in the system between 1200°C and 1400°C.

## 3. Thermodynamic Calculation

**Figure 2:**
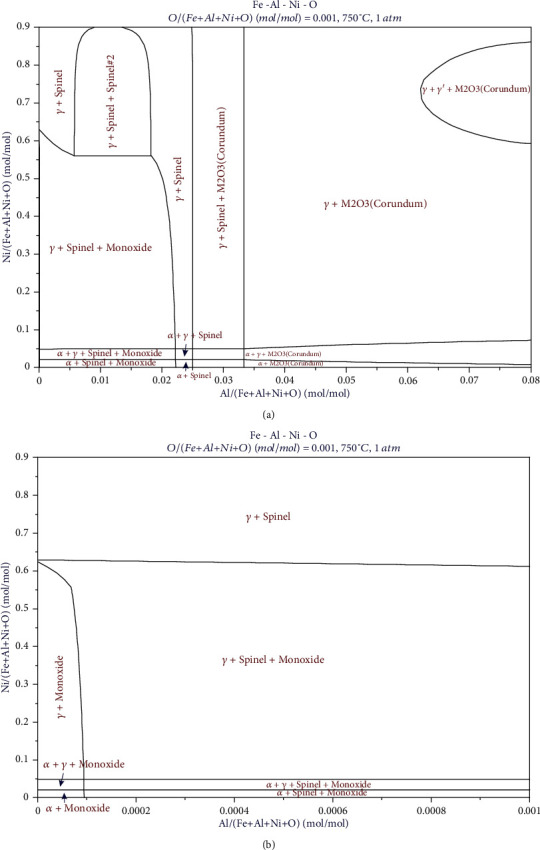
The calculated isothermal section of Fe-Al-Ni-O system at 750°C: (a) Al content ranges from 0 to 8 at.%; (b) Al content ranges from 0 to 0.1 at.%.

In order to have a preliminary understanding of the Fe-Al-Ni-O system and provide a basis for the composition design of the experimental alloys, phase relationship of this system was calculated by using FactSage thermochemical software coupled with FSstel and FToxid databases. In the thermochemical calculation, considering that the oxygen partial pressure of the alloy was extremely low, oxygen content was set extremely low (0.1 at.%), and then, phase relationships of oxidation phase with Al and Ni content were simulated. The results are shown in Figure 2. The oxide coexisting with the matrix alloy phases (*γ*, *α*+*γ*, or *α*) varies with the Al content. Single oxide, monoxide, spinel, and corundum, is in equilibrium with the alloy phases when Al content lies in the ranges of 0~0.01 at.%, 2.2%~2.5 at.%, and more than 3.3 at.%, respectively. Two oxides, spinel and corundum, coexist with the alloy phases when the Al content varies between 2.5 at.% and 3.3 at.%. And monoxide and spinel are in equilibrium with the alloy phases when the Al content is between 0.01 and 2.2 at.%.

## 4. Experimental Procedure

According to the calculated the Fe-Al-Ni-O phase diagram, a series of alloys with Al content less than 5 at.% were prepared to study the oxide boundary; some alloys were added according to the experimental results. 54 Fe-Al-Ni-O specimens in all with the aggregate of 1 g in each were prepared from the Fe powders, Al powders, Ni powders, and Ni_2_O_3_ powders (>99.99%, mass fraction). In this work, the oxygen partial pressure was provided by adding Ni_2_O_3_ (0.02 g) to the sample. Agate mortar was used to mix and grind raw materials evenly to ensure their direct contact. Then, the raw materials were pressed into pieces by a tablet press. After that, the sample was sealed in a corundum crucible and stored in a silicon tube. Then, the silica tube was evacuated and flushed with Ar several times and finally sealed under vacuum. All samples were annealed at 750°C and kept for 45 days. During the annealing process, the Ni_2_O_3_ powders reacted with metal powders to reach the equilibrium between alloy and oxides. In fact, the equilibrium oxygen partial pressure of each sample was different, and the time required for the alloy-oxide system to reach equilibrium was determined by many attempts. It was found that annealing for 45 days could make the sample reach equilibrium. Finally, the samples were rapidly quenched into cold water.

A JSM-6510 scanning electron microscope (SEM) equipped with an Oxford INCA energy-dispersive X-ray spectroscope (EDS) was used to carry out detailed metallographic examination and composition analyses of the unetched samples. The compositions reported here were the average of at least five measurements. In addition, X-ray diffraction analysis of some critical alloys was carried out by using a D/max 2500 PC X-ray diffractometer with Cu K radiation and a step increase of 0.02° in the 2*θ* angle. Si powders were used as external calibrated standard. The XRD patterns were indexed and calculated by Jade software package.

## 5. Result and Discussion

After annealing at 750°C for 45 days, all oxides and corresponding matrix alloy phases observed in the specimens were analyzed. Sixteen key alloys (A1-A8, B1-B8) close to the boundary of corundum and spinel in the 750°C isothermal section of the Fe-Al-Ni-O system were selected. Nominal compositions of these selected specimens are listed in Table 2. According to the oxide phases summarized in Table 1, the corundum oxide phase can be Al_2_O_3_, Fe_2_O_3_, and other oxides, and the spinel oxide phase can be Fe_3_O_4_, FeAl_2_O_4_, NiAl_2_O_4_, and NiFe_2_O_4_. Therefore, spinel can be represented by chemical formula (Fe, Ni) (Al, Fe)_2_O_4_.

### 5.1. Corundum

**Figure 3:**
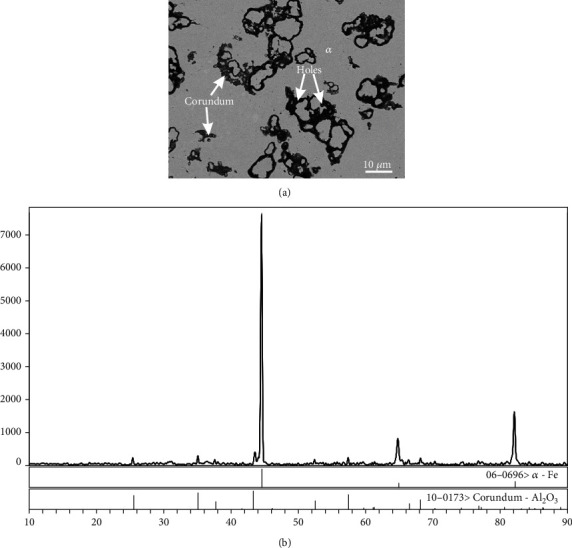
(a) BSE image and (b) XRD pattern of alloy A1 showing the coexistence of the *α* and corundum phases.

The corundum and corresponding matrix phases in alloys A1-A8 identified by a combination of XRD and SEM-EDS are summarized in Table 3. As shown in Figure 3(a), the back-scattered electron (BSE) image of the alloy A1 proved that corundum can be in equilibrium with *α* matrix. The solubility of Fe and Ni in corundum was 1.4 and 3.7 at.%, respectively. The corresponding XRD patterns of the alloy A1 are shown in Figure 3(b). According to SEM and EDS analyses, the black areas in Figure 3(a) were confirmed as holes.

**Figure 4:**
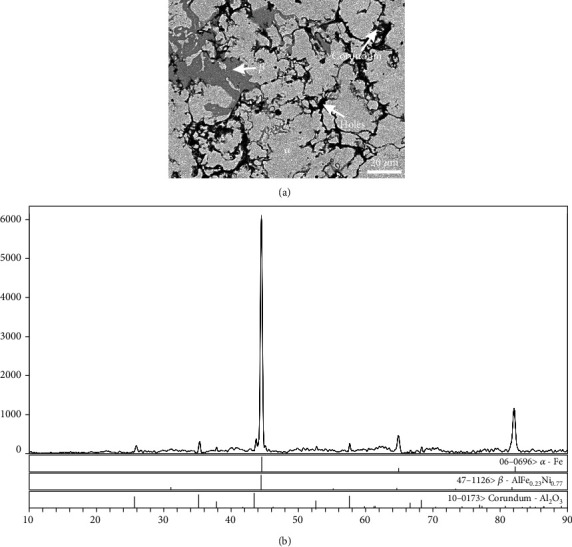
The *α*, *β*, and corundum phases coexisting in alloy A2: (a) BSE image and (b) XRD pattern.

Figure 4(a) shows the BSE image of alloy A2. The light-grey area represents the *α* phase, the dark-grey area stands for the *β* phase, and the less dense area entrapped round holes are the corundum phase. The XRD pattern is shown in Figure 4(b), in which the characteristic peaks of *α*, *β*, and corundum phases are obvious.

**Figure 5:**
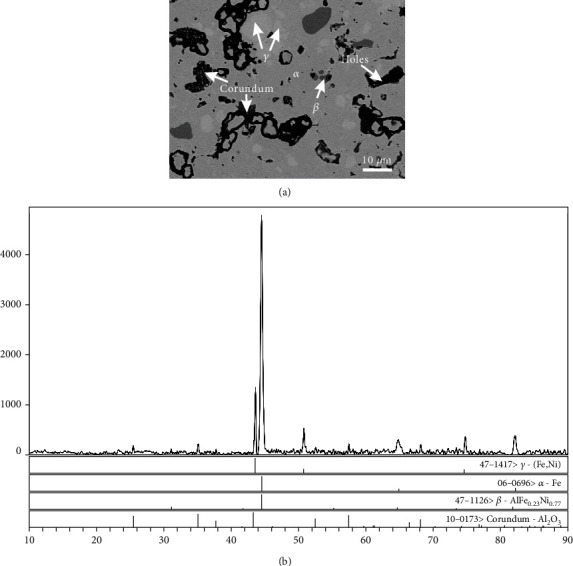
The *α*, *β*, *γ*, and corundum phases coexisting in alloy A3: (a) BSE image and (b) XRD pattern.

Alloy A3 is in the region of four phases, *α*, *β*, *γ*, and corundum, as shown in Figure 5(a), in which the dark grey phase containing 32.3 at.% Al, 30.2 at.% Fe, and 37.5 at.% Ni is the *β* phase. Although the Al content in the *α* phase (8.9 at.% Al, 85.2 at.% Fe, and 5.9 at.% Ni) gets close to that in the *γ* phase (8.6 at.% Al, 76.1 at.% Fe, and 15.3 at.% Ni), the *α* and *γ* phases can be distinguished by changing contrast and brightness. As shown in Figure 5(b), characteristic peaks of both *α* and *γ* are evidenced in the XRD patterns of alloy A3.

**Figure 6:**
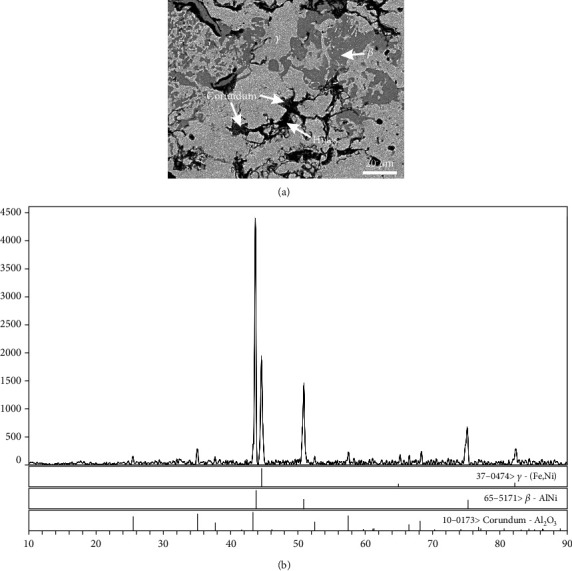
(a) BSE image and (b) XRD pattern of alloy A4 revealing that the *β*, *γ*, and corundum phases were in equilibrium.

As seen from Figure 6(a), three-phase equilibrium between *β*, *γ*, and corundum was found in alloy A4. As the average atomic weight of the *γ* phase (Al: 8.4 at.%; Fe: 66.4 at.%; Ni: 25.2 at.%) is more than that of the *β* phase (Al: 31.3 at.%; Fe: 30.8 at.%; Ni: 37.9 at.%), the light grey phase is *γ* and the dark grey phase is *β* based on the image contrast. And the porous corundum phase is situated around the boundary of holes. Three-phase equilibrium of the above three phases in alloy A4 was confirmed by XRD patterns in Figure 6(b). It is worth mentioning that although the crystal structure types of the *β* phase in alloys A2, A3, and A4 are the same, the selected PDFs are different due to the varied solubility of elements and the change of lattice constants. The same is true for the *γ* phase in alloys A3 and A4.

**Figure 7:**
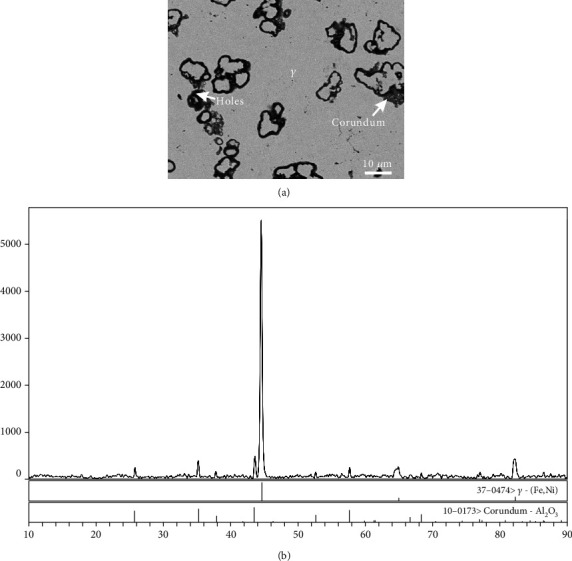
The *γ* and corundum phases were in equilibrium in alloy A5: (a) BSE image and (b) XRD pattern.

The phase equilibrium between *γ* and corundum has been evidenced in alloys A5 to A8. As shown in Figure 7(a), in which the grey matrix phase is the *γ* phase, the dark grey phase located around the boundary of holes is the corundum phase according to the EDS analysis. The XRD patterns are shown in Figure 7(b), in which the characteristic peaks of the above two phases are proved. Based on the analyses of EDS and XRD patterns, corundum is mainly Al_2_O_3_.

In the Fe-Al-Ni system, Al has a stronger affinity for oxygen than Fe and Ni, and Al_2_O_3_ has the largest negative value for Gibbs free energy of formation than FeO and FeAl_2_O_4_, so Al_2_O_3_ is the most stable oxide in the Fe-Al-Ni-O system at low oxygen pressure. According to selective oxidation theory of Wagner [28, 29], when the Al concentration is lower than a critical value for the transformation from internal oxidation to external oxidation, Al_2_O_3_ will be formed in the subsurface layer of the specimen. Meanwhile, the Pilling-Bedworth ratio of Al_2_O_3_ is 1.29 [30], indicating a higher volume of the oxide than the volume of the metal, so there will be large stress produced during oxide's formation. That is account for why the corundum phase always locates around the boundary of holes.

### 5.2. Spinel

**Figure 8:**
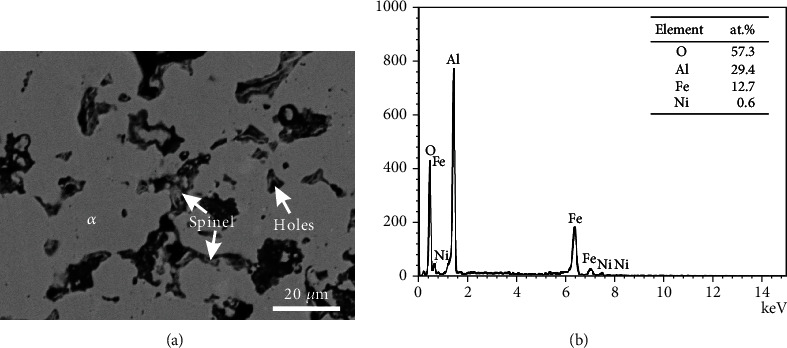
(a) BSE image and (b) the EDS patterns of the spinel phase in alloy B1.

**Figure 9:**
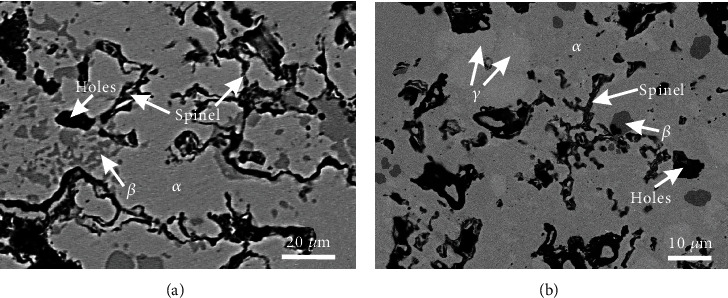
BSE images of (a) alloy B2 and (b) alloy B3.

**Figure 10:**
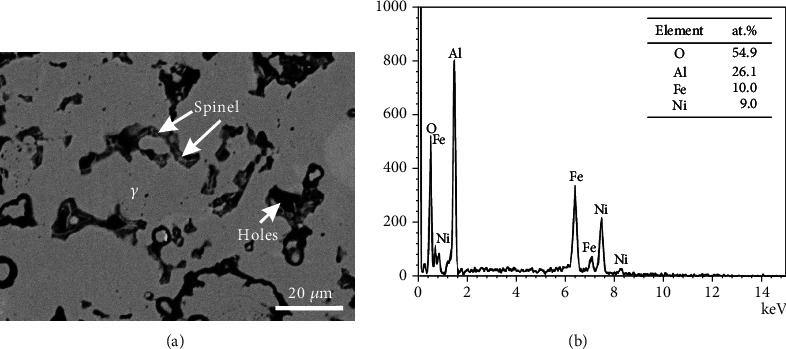
(a) BSE image and (b) the EDS patterns of the spinel phase in alloy B6.

Table 4 shows the oxide phase spinel and the corresponding matrix phases detected in alloys B1-B8.  Figure 8(a) is the BSE micrographs of the alloy B1. The matrix phase *α* is the same as above in Figure 3(a), and there are fine oxide particles around the grain boundary of the *α* phase. As the amount of these fine particles is low, it is difficult to identify them by XRD. However, the EDS pattern shown in Figure 8(b) suggests that it is the spinel phase. As being shown in Figure 9(a), the alloy B2 is located in the region of three phases, *α*, *β*, and spinel. BSE image shown in Figure 9(b) illustrates that the alloy B3 lies in the four-phase region, *α*, *β*, *γ*, and spinel. As same as that in the alloy B1, the spinel phase in B2-B3 was also determined based on EDS patterns. SEM-EDS analysis indicates that the alloys B4-B8 locate in the same two-phase region: *γ* and spinel. Typical micrograph of the alloy B6 is shown in Figure 10(a), and EDS patterns of the spinel phase are shown in Figure 10(b). By comparing Figure 10(b) with Figure 8(b), it can be found that the characteristic peaks of Ni in Figure 10(b) are much more obvious, indicating Ni content in the spinel phase in the alloy B6 is higher than that in the alloy B1.

### 5.3. Discussion

**Figure 11:**
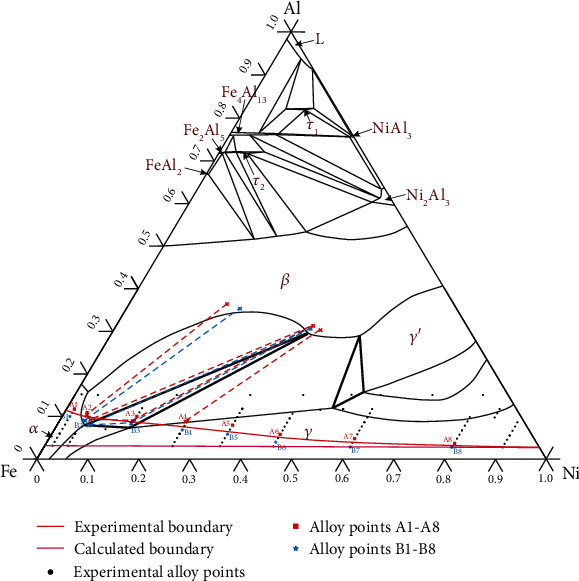
Phase boundary between corundum and spinel oxides in the 750°C isothermal section of the Fe-Al-Ni-O system.

According to the experimental results analyzed above, the 750°C isothermal section of the Fe-Al-Ni-O system is constructed in a combination of the calculated Fe-Al-Ni phase diagram and experimentally detected oxide boundary between corundum and spinel. This isothermal section, also named primary oxide phase diagram of the Fe-Al-Ni-O system at 750°C, is shown in Figure 11. The following 9 regions are confirmed: (1) *α*+corundum, (2) *α*+*β*+corundum, (3) *α*+*β*+*γ*+corundum, (4) *β*+*γ*+corundum, (5) *γ*+corundum, (6) *α*+spinel, (7) *α*+*β*+spinel, (8) *α*+*β*+*γ*+spinel, and (9) *γ*+spinel. The boundary between corundum and spinel measured by experiment is represented by a red line, and the calculated boundary is represented by a purple line. Red-dot lines are tie lines of the matrix alloy phases coexisting with corundum, and blue-dot lines are tie lines of the matrix alloy phases coexisting with spinel. It is worth mentioning that there should be a region where corundum and spinel coexist with the matrix alloy phase, for example, *γ*+corundum+spinel three-phase region in Figure 2(a). However, because the amount of oxide is too small to be analyzed by XRD, and the composition range for this region is limited; only the boundary between corundum and spinel is indicated in this study. Compared with the calculated results, the oxide phase boundary obtained in this experiment obviously moves to the Ni-Al side. When the Fe content is high, the experimental boundary offsets to the Al side. With the increase of Ni content, the boundary will gradually approach the calculated boundary and eventually almost overlap.

From the experimental phase diagram, the spinel oxide can be formed only when the composition points of the original alloy fall in the region of phase equilibrium between spinel and matrix. Meanwhile, due to the low Gibbs free energy of alumina and the low equilibrium oxygen pressure required for the formation of the spinel phase, internal oxidation is easy to occur when the Al content is low. According to Wagner's oxidation theory [28], the transition of spinel phase from internal oxidation to external oxidation to form a continuous spinel film requires the Al content to reach a critical value. Therefore, when designing the alloy composition in aim of forming anticorrosion spinel, both the phase equilibrium and the critical Al content under different oxygen pressure should be considered.

## 6. Conclusion

Phase equilibria of the Fe-Al-Ni-O system at 750°C were determined using the equilibration and quenching techniques, followed by characterization of alloys by means of SEM-EDS and XRD. Corundum can equilibrate with alloys close to the Al-rich region. Spinel is in equilibrium with matrix alloy phases close to the Fe-Ni side. Compared with the calculated results, the phase boundary of oxides moves to the Ni-Al side partially. Two four-phase equilibrium regions, *α*+*β*+*γ*+corundum and *α*+*β*+*γ*+spinel, and three three-phase equilibrium regions, *α*+*β*+corundum, *β*+*γ*+corundum, and *α*+*β*+spinel, were confirmed. This primary oxide phase diagram can be used as a guide for the preparation of the oxide film by selective oxidation.

## Figures and Tables

**Table 1:** Crystal structure data for the matrix phases and oxides in the Fe-Al-Ni-O system.

Phase	Pearson symbol	Space group	Lattice parameters (nm)			PDF no.
			*a*	*b*	*c*	
*α*-Fe	*cI2*	*Im3m*	2.866	2.866	2.866	06-0696
*γ*-(Fe,Ni)	*cI2*	*Im3m*	2.868	2.868	2.868	37-0474
*γ*-(Fe,Ni)	*cF4*	*Fm3m*	3.598	3.598	3.598	47-1417
*β*-FeAl	*cP2*	*Pm3m*	2.881	2.881	2.881	33-0020
*β*-NiAl	*cP2*	*Pm3m*	2.910	2.910	2.910	65-5171
*β*-AlFe_0.23_Ni_0.77_	*cP2*	*Pm3m*	2.885	2.885	2.885	47-1126
*γ*′-Ni_3_Al	*cP4*	*Pm3m*	3.572	3.572	3.572	65-6613
NiAl_3_	*oP16*	*Parma*	6.598	7.352	4.802	02-0416
Ni_2_Al_3_	*hP5*	*P3m1*	4.036	4.036	4.900	65-3454
Fe_4_Al_13_	*mC102*	*C2/m*	15.492	8.078	12.471	50-0797
Fe_2_Al_5_	*oC14*	*Cmcm*	7.649	6.413	4.216	47-1435
FeAl_2_	*aP18*	*P1*	4.878	6.461	8.748	33-0019
FeAl_9_Ni	*mp22*	*P2_1_/c*	8.598	6.271	6.207	[16]
Fe_3_Al_10_Ni	*hP28*	*P6_3_/mmc*	_	_	_	[16]
Al_2_O_3_ (corundum structure)	*hR10*	*R3C*	4.758	4.758	12.991	10-0173
Fe_2_O_3_ (corundum structure)	*hR10*	*R3C*	5.036	5.036	13.749	33-0664
Ni_2_O_3_ (corundum structure)	*hp10*	*P*	4.610	4.610	5.610	14-0481
FeAlO_3_ (corundum structure)	*oP40*	*Pc21n*(33)	8.566	9.249	4.989	30-0024
FeAl_2_O_4_ (spinel structure)	*cF56*	*Fd3m*	8.153	8.153	8.153	34-0192
NiAl_2_O_4_ (spinel structure)	*cF56*	*Fd3m*	8.048	8.048	8.048	10-0339
Fe_3_O_4_ (spinel structure)	*cF56*	*Fd3m*	8.090	8.090	8.090	65-3107
NiFe_2_O_4_ (spinel structure)	*cF56*	*Fd3m*	8.337	8.337	8.337	54-0964
FeO (NaCl structure)	*cF8*	*Fm3m*	4.293	4.293	4.293	46-1312
NiO (NaCl structure)	*cF8*	*Fm3m*	4.177	4.177	4.177	47-1049

**Table 2:** Nominal compositions (at.%) of typical specimens in this study.

No.	Al	Fe	Ni	No.	Al	Fe	Ni
A1	12	86	2	B1	10	88	2
A2	11	84	5	B2	9	86	5
A3	10	75	15	B3	8	77	15
A4	9	66	25	B4	7	68	25
A5	8	57	35	B5	6	59	35
A6	6	49	45	B6	4	51	45
A7	5	35	60	B7	3	37	60
A8	4	16	80	B8	3	17	80

**Table 3:** Constitution of the corundum and matrix phases coexisting in the Fe-Al-Ni-O system at 750°C.

No.	Substrate				Oxides				
	Al	Fe	Ni	Matrix phases	O	Al	Fe	Ni	Oxide phases
A1	10.8	86.9	2.3	*α*	58.4	36.5	1.4	3.7	Corundum
A2	10.7	85.2	4.1	*α*	59.9	36.2	1.5	2.4	Corundum
	37.8	41.0	21.2	*β*					
A3	8.9	85.2	5.9	*α*	60.6	35.8	1.7	1.9	Corundum
	32.3	30.2	37.5	*β*					
	8.6	76.1	15.3	*γ*					
A4	31.3	30.8	37.9	*β*	56.7	36.9	2.3	4.1	Corundum
	8.4	66.4	25.2	*γ*					
A5	7.6	54.9	37.5	*γ*	57.9	36.4	2.2	3.5	Corundum
A6	5.7	49.5	44.8	*γ*	59.5	37.1	0.3	3.1	Corundum
A7	4.1	35.1	60.8	*γ*	56.2	37.0	1.4	5.4	Corundum
A8	3.7	17.4	78.9	*γ*	61.2	35.0	0.3	3.5	Corundum

**Table 4:** Constitution of the spinel and matrix phases coexisting in the Fe-Al-Ni-O system at 750°C.

No.	Substrate				Oxides				
	Al	Fe	Ni	Matrix phases	O	Al	Fe	Ni	Oxide phases
B1	9.8	88.1	2.1	*α*	57.3	29.4	12.7	0.6	Spinel
B2	8.8	86.6	4.6	*α*	58.3	27.8	12.1	1.8	Spinel
	36.9	39.8	23.3	*β*					
B3	7.5	86.4	6.1	*α*	57.0	27.8	11.1	4.1	Spinel
	31.2	31.1	37.7	*β*					
	8.2	76.1	15.7	*γ*					
B4	6.8	68.6	24.6	*γ*	56.0	26.9	10.9	6.2	Spinel
B5	5.5	58.9	35.6	*γ*	56.5	25.6	10.7	7.2	Spinel
B6	3.7	52.1	44.2	*γ*	54.9	26.1	10.0	9.0	Spinel
B7	2.8	38.3	58.9	*γ*	54.7	27.1	10.1	8.1	Spinel
B8	2.7	19.5	77.8	*γ*	56.8	24.9	9.8	8.5	Spinel

## Data Availability

The authors confirm that the data supporting the findings of this study are available within the article.
